# Reactivity of Calcined Clays as SCM—A Review

**DOI:** 10.3390/ma17020312

**Published:** 2024-01-08

**Authors:** Steffen Overmann, Anya Vollpracht, Thomas Matschei

**Affiliations:** Institute of Building Materials Research (ibac), RWTH Aachen University, Schinkelstr. 3, 52062 Aachen, Germany; vollpracht@ibac.rwth-aachen.de (A.V.); matschei@ibac.rwth-aachen.de (T.M.)

**Keywords:** calcined clay, reactivity, relative compressive strength, cement

## Abstract

Calcined mixed clays are one of the most promising alternative supplementary cementitious materials. However, their standardized use is difficult due to the wide range of compositions of the raw materials. The reactivity potential of different clays can hardly be estimated on the basis of simple characteristics so far. This review aims to identify and compile the factors that determine reactivity. At first, an overview of the methods to evaluate reactivity is presented in order to provide a definition of this term. Subsequently, the reactivity-determining factors are compiled and subdivided into the characteristics of the raw material (chemical and mineralogical composition), the parameters of calcination (furnace type, temperature, grain size, retention time, and cooling), and the characteristics of the calcined material (physical properties and amorphous phase). Interrelations are discussed qualitatively. In the second step, a quantitative literature analysis was conducted to quantify correlations between the different factors and reactivity. However, since the characterization methods in the literature are very different, the data can hardly be analyzed quantitatively. Consequently, this paper points out what information is needed to conduct profound, comparable studies to evaluate the reactivity potential of clays.

## 1. Introduction

The cement industry is known to contribute significantly to global CO_2_ emissions due to the production of Portland cement clinker. The CO_2_ emissions and energy balance of concrete are largely determined by the portion of Portland cement clinker in the binder. Hence, the use of cement substitutes, so-called supplementary cementitious materials (SCM), is of great importance for cement/concrete production. Besides the ecological factor, the technical requirements of novel concrete formulations are increasing, for example, to fulfill related durability criteria [1]. Therefore, the binders have to be adapted to these requirements by the use of SCMs or alternative binder systems. Moreover, due to further environmental efforts, Germany has planned to switch to renewable energy generation. Thus, it planned the gradual shutdown of all coal-fired plants until 2038 [2]. As a result, the availability of the second-most widely used SCM in Germany, fly ash, is drastically decreasing.

Calcined clays are an ecologically and economically interesting alternative SCM. The clays, as raw materials, are available worldwide and have lower CO_2_ emissions during calcination due to their lower lime content and lower temperature treatment compared to cement clinker. The composition of raw clays can be very complex with a mixture of different phyllosilicates such as kaolinite, smectite, illite, and mica, as well as various other components such as quartz, feldspar, iron oxides, carbonates, sulfates, and iron sulfides [3].

A well-known clay-based SCM is the highly reactive metakaolin, which is produced from kaolin, a claystone that contains a high content of the clay-mineral kaolinite. It has been the subject of many studies, e.g., [4,5,6,7,8]. A broad application in the cement/concrete industry is currently impeded by its high price, caused by the limited availability, the costly purification process, and the high demand by competing industries such as the ceramic or paper industry [9,10]. Furthermore, due to the very high reactivity in terms of portlandite consumption, it can only be used in a limited proportion, similar to silica fume.

Therefore, the less pure and widespread common clays have been the focus of research for around three decades [11,12,13] with steadily increasing relevance. Feasibility studies around the world are summarized e.g., in [14,15,16]. It can be concluded that, although the reactivity of each clay is very different, the clays mostly are suitable as SCM in their activated state. Even illitic clays, which are ascribed to have a rather low potential compared to other clays (see Section 4.1.1), are suitable to produce geopolymers, which serve as an alternative binder system that does not use Portland cement [17]. However, many studies showed that for the different clay-mineral phases, the optimum calcination temperature varies, e.g., [18,19,20,21,22]. In addition, for the same clay minerals, the optimum calcination temperature varies from one study to another. In addition, the mineral phases differ in their reactivity potential [18,19,20,21,22]. Furthermore, accessory minerals can influence the reactions during calcination [22,23,24]. In order to identify strategies for their use as standardized SCM, this review compiles the effects on reactivity of calcined clays. Since other literature on the subject of calcined clays deals just with individual aspects with regard to the reactivity-determining factors, this study is intended to provide a comprehensive overview of the state-of-the-art. However, many properties influence reactivity, and there are various characterization methods, parameters to be measured, and approaches for investigating the reactivity behavior. Therefore, after a brief overview of the basics of clay mineralogy, the methods for investigating the reactivity of clays are introduced. The focus of the study is then on the compilation and evaluation of the reactivity-determining factors in a qualitative and quantitative approach. Due to the complexity of the subject, only the reactivity of calcined clays in the binary cement system is considered, which forms the basis for the understanding of more complex systems, e.g., limestone-calcined clay systems.

## 2. Clay—An Overview

There is no uniform definition of the term “clay.” However, from a geological perspective, clays are in general unconsolidated sedimentary rocks or in a consolidated form referred to as clay stones, which are composed predominantly of very fine-grained silicate minerals. According to EN ISO 14688–1 clay is defined by the grain size of <2 µm. In terms of quantity, clay minerals compose the largest proportion. However, the term “clay” is used here for all sediments containing clay minerals independent of the total content. Depending on the grain size distribution, some of the “low-grade clays” would rather be classified as “loam.” Clay minerals have a lamellar structure with particle sizes mainly smaller than 2 μm and are categorized as phyllosilicates. Their wide availability is due to the fact that they mainly arise from the weathering of silicate rocks [3,25].

The following section provides a basic overview of clay mineralogy. However, as the clay mineralogy is very diverse and complex, more detailed information can be found e.g., in [26].

**Figure 1:**
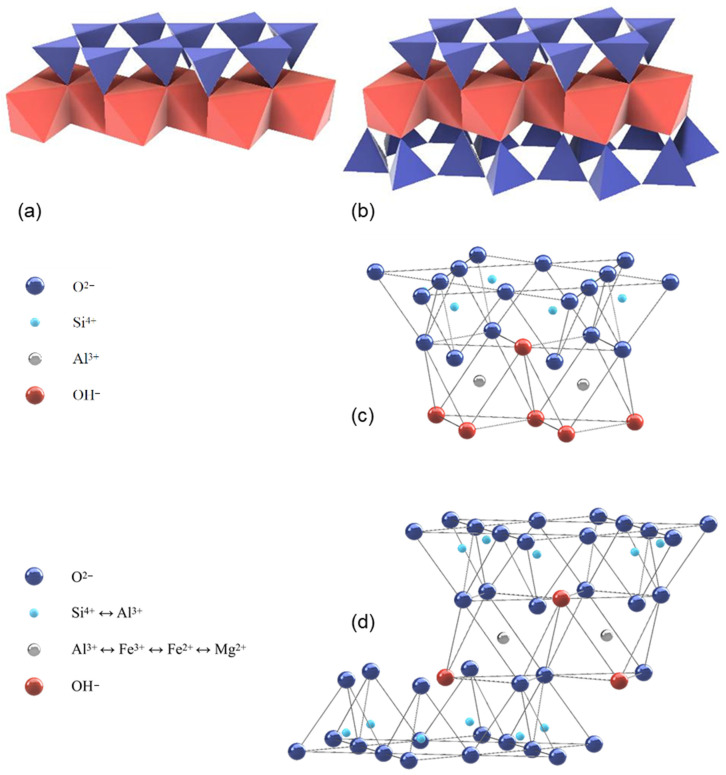
1:1 layer structure e.g., kaolinite (**a**) and 2:1 layer structure e.g., montmorillonite (**b**) illustrated with tetrahedra and octahedra (modified after [26]); 1:1 layer structure of kaolinite (**c**) and 2:1 layer structure of illite (**d**) illustrated with atom positions (modified after [27]).

The clay minerals ideally contain a continuous sheet of SiO_4_ tetrahedrons, which are linked to the adjacent ones over three corners to a planar pseudo-hexagonal structure, while the free corners of each tetrahedron point to the same side of the sheet (see Figure 1).

These corners are linked to octahedrons with edges of OH^−^, which are partly substituted by sharing oxygen bonds. For the non-shared corners of the octahedrons, an occupation with F^−^ and Cl^−^ is also possible. The OH^−^ octahedrons are also forming a layer with connections between each other by sharing edges. In the tetrahedrons, Si^4+^ can be substituted by Al^3+^ or Fe^3+^ as tetrahedral cations. Octahedral cations are usually Al^3+^, Fe^3+^, Mg^2+^, and Fe^2+^ [25,26] (see Figure 1). Depending on the number of cations at the octahedral centers, the phyllosilicates are designated as di- (occupation of all octahedral centers by divalent cations) or as trioctahedral (2/3s of the octahedral sites are occupied by trivalent cations).

The lateral dimension of the tetrahedral sheet is usually slightly wider than that of the octahedral sheet. This misfit needs to be adjusted by the distorting or bending of one or both layers. This distortion leads to a displacement of the ideal hexagonal symmetry to low triclinic or monoclinic symmetry [26].

The structure of the clay minerals consists of certain sequences of mostly the same elementary layers, which are separated by intermediate layers. The 1:1 layer structure (two-layer minerals such as kaolinite) consists of the repetition of one tetrahedral and one octahedral sheet as the elementary layer, while in the 2:1 layer structure (three-layer minerals such as montmorillonite or illite), one octahedral sheet is sandwiched between two tetrahedral sheets as the elementary layer. The two-layer minerals ensure the cohesion of the elementary layers via hydrogen bridges between the outer oxygen ions of the tetrahedral layers and the hydroxide ions of the opposing octahedral layers. For three-layer minerals, only oxygen ions are situated opposite each other, which means that the cohesion initially is realized exclusively by van der Waals forces. However, for three-layer minerals with a negative layer charge, the elementary layers are connected additionally through the incorporation of cations, mostly large alkali or alkaline earth ions, which ensure a charge balance in the intermediate layer. Furthermore, there are four-layer (or 2:1:1) clay minerals such as chlorite. The general structure consists of a 2:1 structure, plus a sheet of octahedrally coordinated cations, in the interlayer. A general overview of the characteristics and properties of uncalcined clays can be found e.g., in [28]. In the following, the ideal structure of the most common clay minerals (kaolinite, montmorillonite, and illite) are more closely examined [21].

### 2.1. Kaolinite

Kaolinite is a dioctrahedral two-layer mineral where the octahedron gaps are exclusively occupied with aluminum. All tetrahedral gaps are occupied with Si^4+^. The general stoichiometric composition is Al_2_Si_2_O_5_(OH)_4_. There are usually very few substitutions. Due to the lack of layer charge, no cations are bound in the intermediate layer, and the cohesion is caused by hydrogen bonds between the elementary layers [27].

### 2.2. Montmorillonite

Montmorillonite is the most important representative of the smectites, a group of swellable 2:1 layered alumosilicates. It has layer charges of around −0.2 to −0.6 [29] and has a dioctahedral occupancy with Al^3+^ ions. The charge is located predominantly in the octahedron layer, where aluminum ions are partially replaced by divalent cations (mostly Mg^2+^). The charge is balanced by alkali or alkaline earth ions in the intermediate layer. The structural formula is ($M_{y}^{+}$ × nH_2_O)(${Al}_{2-y}^{3+}$ ${Mg}_{y}^{2+}$)_2_${Si}_{4}^{4+}$O_10_(OH)_2_ [26]. In contrast to the non-swelling illites, montmorillonite is swellable due to the lower layer charge caused by the lower number of substitutions. The electrostatic forces binding interlayer cations to the crystal layers are of the same order of magnitude as the hydration energy of the interlayer cations. Therefore, strongly hydrating cations such as Na^+^, Ca^2+^, and Mg^2+^ can draw water molecules into the interlayer [30].

### 2.3. Illite/Muscovite

Illite is a three-layer phyllosilicate based on the muscovite structure as a weathering product of muscovite but with diverse possibilities of substitutions. Muscovite is a three-layer phyllosilicate with a very stable structure. Every fourth tetrahedron is occupied by Al^3+^ instead of Si^4+^. The occurring layer charge is compensated by the incorporation of K^+^ in the intermediate layer in the area of the hexagonal gaps in the tetrahedral sheets. The term “illite” is used for 2:1 minerals with non-expandable layers and a wide variety of chemical compositions. During the weathering of muscovite, K^+^ emigrates from the intermediate layer and is partly substituted by other cations such as Ca^2+^ and Na^+^, while in the tetrahedral layer, Si^4+^ is partly substituted by Al^3+^. In the octahedral layers, Al^3+^ is substituted by Fe^3+^, Mg^2+^, and Fe^2+^ [25]. The layer charge is between −0.6 and −0.9 [26]. Due to this higher layer charge compared to smectites, illites are not notably swellable.

## 3. Methods to Investigate the Reactivity of Calcined Clays

Reactivity is considered here as the availability of Si and Al from the clay in a cementitious environment and the contribution of these to the formation of strength-building phases.

A lot of different test methods to evaluate the reactivity of SCMs with different approaches can be found in literature as compiled in Table 1 (compare [31]).

In general, the reactivity tests are intended to enable a prediction of strength development in a cement system as this is the application scenario. So, most of the authors are also using relative compressive strength tests (different cement substitution levels relative to 100 wt.% cement) of cement pastes, mortars, or concretes for evaluating the performance of calcined clays. However, when using different cements and mix compositions in the relative strength tests, the reactivity of calcined clays will be manifested in varying degrees, resulting in problems with comparability. For example, when using the same cement type, a higher alkali content will result in higher relative compressive strengths [21].

## 4. Qualitative Literature Evaluation Regarding the Reactivity-Determining Factors

### 4.1. Characteristics of the Raw Material

#### 4.1.1. Mineral Phase Composition

##### General Remarks

**Figure 2:**
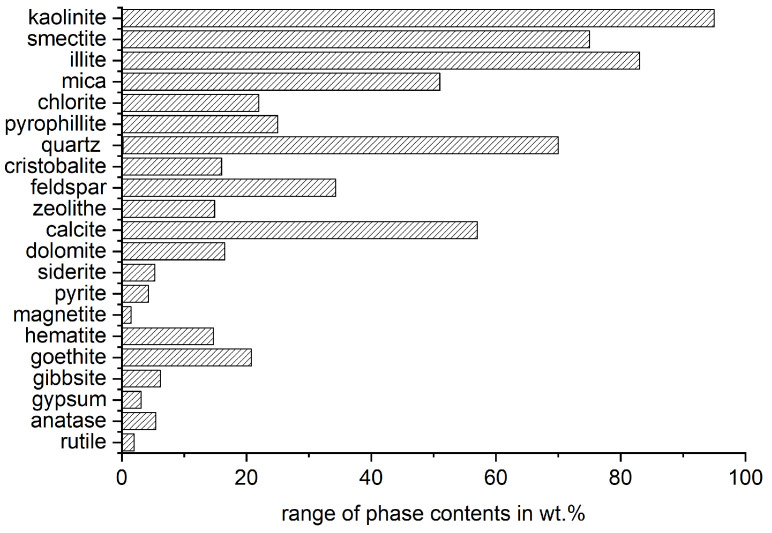
Range of phase contents of raw clays from literature (see electronic File S1).

The most influencing characteristic of the reactivity potential of clays is the mineral phase composition of the raw material. Concerning an appropriate mineralogical phase characterization, it is referred to [60]. Firstly, the material must contain sufficient content of activatable phases. Lopez [61] found that a kaolinite content in the raw clay of 40 wt.% is already enough to maintain or increase the mortar strength at 28 d with a substitution level of the cement of 30 wt.%. However, the required content of activatable phases depends on the type of clay mineral and, of course, the intended substitution level and application of the binder. Figure 2 shows the range of mineral phases in clays that was compiled from the literature for Section 5 and illustrates the complexity of these materials.

As the phase composition varies over a wide range, and different phases with different reactivity potential are occurring together, at the current state of the art, it is not possible to directly predict the reactivity potential of mixed clays just according to the mineral composition.

##### Clay-Mineral Phases

Many researchers found the order of reaction potential of the most common clay-mineral phases to be as follows: kaolinite > smectite (Ca–montmorillonite > Na–montmorillonite) > illite [12,18,19,20,21,22,62,63]. The binding conditions of Al and Si defined by the crystal structures of the different phases determine how easily these phases can be activated by thermal treatment, as well as the potential release of Al and Si in the cementitious environment. Fernandez et al. [20] stated that the higher reactivity potential of kaolinite, in comparison to the other clay minerals, originates from its higher content of hydroxyl groups and their location in the crystal structure where they coordinate Al to a larger amount. During the dehydroxylation process, this leads to more disorder and a greater exposure of Al groups at the surface of the phases. In comparison, illite and montmorillonite seem to conserve the order of their structural layers, even after complete dehydroxylation. Furthermore, Al groups are trapped between silicate tetrahedrons and are less available for the reaction. Thus, it is more difficult to break down the silica or alumina–silica networks of these clay types in an alkaline media.

For kaolinitic clays, Scrivener et al. [64] showed that the secondary phases, e.g., quartz and other clay minerals, do not have a significant impact on the compressive strength, and the kaolinite content is the dominating factor. A similar result was found by Avet et al., 2016 [65]. However, it is not clear how dominant the kaolinite content is when other clay phases have a significantly higher proportion. Bratoev et al. [66] investigated eight different calcined clays and found that the reactivity (with a Chapelle test) mainly depends on the contents of kaolinite and montmorillonite in the raw clay and quantified their contribution to reactivity. They scaled the consumed Ca(OH)_2_ to the kaolinite content and the montmorillonite content, respectively, by investigating two clays, one with kaolinite as its single clay phase and the other with montmorillonite. They found a consumption of 12.4 mg and 5.9 mg of calcium hydroxide for every wt.% of kaolinite and montmorillonite, respectively, and they could recalculate the consumption of other mixed clays (also containing illite) quite well. However, for illite activation, the calcination temperature of 800 °C in these experiments seems to be too low (see Section 4.2.2).

Besides the effect of different clay mineral groups, the degree of crystallinity or lattice disorder is a potential factor for the activation of clay minerals. Studies from the literature showed that well-ordered kaolinites stay stable longer during the temperature treatment. Afterward, they show less pozzolanic reactivity than poorly ordered ones [12,39,67]. This can be explained by the lattice defects, which weaken the bonds. Thus, less energy is required to break them. Kaolinite, especially, is known to occur often in low crystallinity. This is assumed to be caused by randomly distributed Al in the octahedral position, the substitution of Al by Fe or Ti, and the occasional interlayer water between the silicate units [27].

The literature on less frequent clay minerals used as SCM in a calcined state is rare as these minerals are usually not predominant in the clays. However, there are studies on the pozzolanic reactivity of halloysite, a mineral from the kaolinite group [68,69]. Although halloysite shows good pozzolanic potential, its importance as an SCM is minor due to its rare occurrence.

##### Non-Clay Phases, including Limestone

Besides the dilution effects of non-clay phases on the reactive binder component, some can also react and have a positive effect on the performance of the material. The most well-known aspect of many studies in the literature is the synergy of calcined clay in combination with limestone in the cement system. The alumina from the clay can react with the limestone and form carbo–aluminate hydrates, which contribute to strength and durability [70]. However, in this review, the combination of calcined clay with limestone is not the focus. A reference is made to Sharma et al. [71] for this aspect.

However, in nature, the paragenesis of clay and calcite, so-called marl, is abundant. If marl is calcined to produce an SCM, the calcite is also exposed to the calcination process where it can decompose at temperatures above 750 °C [72]. Danner [22] replaced 20 wt.% of kaolinitic clay with calcite before and after calcination at different temperatures up to 850 °C. He found no significant influence on the reactivity (relative compressive strength), regardless of whether the calcite is partially decomposed during the calcination or not. Zunino et al. [73] observed with XRD only traces of free lime after the calcination of a kaolinitic clay with 8 wt.% calcite addition at 800 °C and a resulting calcite content of 2.2 wt.%. With SEM, it was found that CaO builds granular deposits on the metakaolinite as a likely amorphous transition state between the free lime and metakaolinite before the recrystallization of new Ca-bearing phases. However, according to Zunino et al. [73], the deposit on the metakaolin surface reduces the specific surface area and, thus, slightly reduces the reactivity. In contrast, for an illitic and smectitic clay containing 25 wt.% and 15 wt.% calcite, respectively, Danner et al. [74] found that after calcination at 800 °C, an increase in the glassy phase, in comparison to clays with less calcite content, results in a higher reactivity. It can be assumed that the formed Ca-bearing glassy phases are more reactive than the metaphases of illite and montmorillonite but less in comparison to metakaolin. However, Danner [22] also found that the recrystallization temperature can decrease when calcite is completely decomposed. By calcining smectitic marl at 850 °C, after the complete decomposition of calcite and amorphization of smectite, Ca-bearing phases such as anorthite (CaAl_2_Si_2_O_8_) and wollastonite (CaSiO_3_) recrystallized and reduced the reactivity. It can be concluded that the decomposition of calcite during the calcination process lowers the melting and recrystallization temperature.

As found by Bullerjahn et al. [23], dolomite (CaMg[CO_3_]_2_) co-calcination can also lower the melting temperature and improve the reactivity of calcined clays. The arising reactive lime and periclase during calcination reacts with the decomposed aluminosilicates, as well as with some feldspar and silica phases forming reactive (poorly) crystalline phases such as melilite types, calcium aluminates, and a vitreous phase. The reaction products in the cement system also differ from those with calcined neat clays.

The effect of iron sulfides (up to 20 wt.% substitution of kaolinite by troilite and pyrite) as other decomposing components potentially occurring in clays on the properties of the calcined clays was investigated by Zunino and Scrivener [75]. It was found that the sulfides completely decompose during calcination, forming hematite and having no effect on reactivity.

Ghorbel and Samet [76] enriched kaolinite with hematite, and goethite precipitated on the kaolinite sheets prior to calcination, and found that the reactivity (portlandite consumption and relative compressive strength) was enhanced until an optimum iron content as iron entered into CSH and ettringite. With iron contents beyond the optimum, the formation of a new iron-bearing phase in the kaolinite–white cement system was observed. As hematite is known to be a very temperature-stable mineral and was still observed in the calcined clay, it can be assumed that goethite has caused the described increase in reactivity.

Furthermore, considered “inert” phases can also contribute to the reactivity of the cementitious system. Danner [22] found that, due to the potentially high surface area of quartz and feldspar formed through weathering processes, these minerals can act as nucleation sites for cement hydration products. It is suggested that this might help to enhance the early-age strength in mortars using blended cements. A similar effect is assumed for iron hydroxides with high specific surface area. Moreover, the reactivity of quartz, feldspars, and zeolithes in a cementitious environment is documented in the literature [77,78,79]. Furthermore, muscovite and K–feldspar in calcined clays can release K ions to the aqueous medium and enhance the pH, which in turn will enhance the pozzolanic reaction [22].

#### 4.1.2. Chemical Composition

Regarding the overall chemical composition of the clay, it does not seem possible to draw conclusions about the pozzolanic potential because Si and Al might also occur in the non-clay phases considered inert, such as quartz and feldspars.

Diaz et al. [80] proposed a pre-evaluation approach for clay deposits based on the Al_2_O_3_ content and the loss on ignition between 350 and 850 °C, which allows for a rough classification between high-kaolinitic, mid-low-kaolinitic, and low-kaolinitic clays with high amounts of 2:1 layer silicates and clays containing appreciable amounts of decomposing non-clay minerals such as carbonates, sulfides, sulfates, and hydroxides. However, this approach is still quite unprecise.

### 4.2. Parameters of Calcination

#### 4.2.1. Calciner Types, Grain Size, and Retention Time

For experiments on a laboratory scale, most authors used muffle furnaces. However, for a higher throughput in an industrial scale, rotary kilns or flash calciners are used. An extensive review of the calcination techniques can be found in [81]. In a rotary kiln, shorter calcination times in comparison to static calcination are possible due to a better dispersion of the particles in the furnace atmosphere. Even less calcination time, in the scale of a few seconds, is required for flash calcination. Just as the optimum calcination time depends on the calciner, the optimum calcination temperature found cannot be transferred between different types of calciners [21,22,82].

Flash calcination also has an effect on the particle texture of metakaolin. Instant dehydroxilation leads to an expansion of the particles and the formation of pores between the clay sheets, leading to a very high specific surface area [21,83]. This also leads to a higher water demand.

The grain size of the clay prior to calcination is also an important parameter for the calcination process because of the temperature gradients occurring in the grains. The volume-to-surface-area ratio defines the kinetics of the calcination progress as reactions propagate from the gas–solid interface. In [82], the amorphous content was found to correlate with particle size as after calcination in a rotary kiln, with input grain sizes up to 100 mm, bigger grains were amorphized to a lower extent. Longer retention times can lower these temperature gradients.

However, Danner [22] and Chakchouk et al. [84] found with lime consumption tests and compressive strength testing, respectively, that longer retention times can also reduce the reactivity, even in a temperature range where no recrystallization occurs. Furthermore, the retention time needed to reach optimum reactivity depends on the temperature, and both parameters depend on the clay mineral phases and their orderly condition, as discussed in Section 4.1.1. According to Bich et al. [85], a more disordered kaolinite structure can dehydroxylate more easily, so the retention time needed is lower.

#### 4.2.2. Calcination Temperature

The optimum calcination temperature for mixed clays is difficult to define as the activation of the different clay minerals is based on different mechanisms. The comparatively low calcination temperature needed for kaolinite compared to illite and montmorillonite is due to the freely accessible OH^−^ ions on the octahedral layer. In the case of three-layer silicates, the OH^−^ ions lie between the SiO_4_ tetrahedral layer and require higher dehydroxilation temperatures [86]. However, as described in Section 4.1.1, after dehydroxilation, the three-layer minerals preserve their crystalline structure, so the amorphization occurs at even higher temperatures through sintering/melting processes, which, in parallel, leads to a reduction in the specific surface area and consequently reduces the reactivity in a physical way [21].

**Figure 3:**
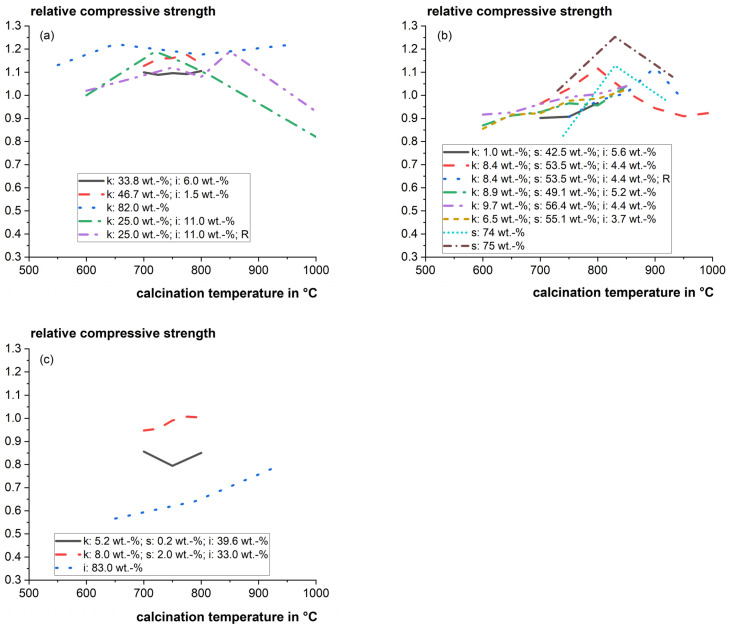
Relative mortar compressive strength as a function of calcination temperature for cement substitution levels of 20–30 wt.% for (**a**) Kaolinite-dominating clays; (**b**) Smectite-dominating clays; and (**c**) Illite-dominating clays (k: kaolinite; s: smectite; i: illite; R: rotary kiln instead of static laboratory furnace) [22,46,47,82,87].

The optimum calcination temperature can be very sensitive to the composition of the clays. Danner [22] found that the reactivity of the investigated calcined smectitic marl changed significantly within a narrow window of 50 °C between incomplete calcination and the beginning of recrystallization. Figure 3 shows a compilation of relative mortar compressive strengths as a function of the calcination temperature for cement substitution levels of 20 to 30 wt.%. The literature data of strength tests strongly vary, and it is difficult to derive a solid general trend for different mixed clays. However, the influence of calcination temperature on the particular main clay phases and their reactivity was studied well in the literature and is discussed in the following sections.

##### Kaolinite

The dehydroxilation of kaolinite induces lattice distortions, resulting in the x-ray amorphous structure of metakaolinite. With a rising degree of dehydroxilation, the degree of amorphization is increasing. The temperature of complete dehydroxilation and amorphization can be found in the literature e.g., around 600 °C [20,21,61,88], 650 °C [46,67], and 700 °C [82], which strongly depends on the crystallinity. In comparison to illite and montmorillonite, in the calcination of kaolinite, NMR studies showed that, in addition to Al^VI^ and Al^IV^, Al^V^ coordination states occur, which demonstrates the significant state of disorder in the metakaolinite [20,21,22,61,89,90]. In the study of Lopez [61], after complete dehydroxylation at 600 °C, the degree of disorder was still rising until 800 °C, which was reflected by increasing Al^V^ coordination states, indicating the disorder is not induced by dehydroxylation alone. Rocha and Klinowski [89] found that, with rising temperatures, Al^VI^ decreases due to the formation of Al^IV^ and Al^V^ until a minimum at 750-to-800 °C, beyond which the Al^VI^ increases again. Torres et al. [90] indicate the maximum disorder in the temperature range of 720–750 °C. At about 900-to-950 °C, a further structural rearrangement occurs, resulting in a defect aluminum–silicon spinel (γ-Al_2_O_3_ type structure). According to Brindley and Nakahira [91], at 925 °C, silicon spinel will be formed; at temperatures > 1100 °C, mullite and cristobalite will be formed. Rocha and Klinowski [89] found, at 950 °C, faint signals of mullite, and at 1000 °C, cristobalite. In summary, at temperatures > 900 °C, first recrystallizations can be expected. Trümer [21] found during the treatment until about 1000 °C that the BET surface varies slightly but stays high, indicating that no sintering or melting occurs. Similar observations were made by Bich et al. [85]. That means metakaolinite does not occur as a real glass as no melting occurs before recrystallization.

Trümer [21] showed with dissolution experiments of Si and Al that for the investigated metakaolin, the optimum calcination temperature with the fastest dissolution kinetic (concentration of dissolved Al and Si after 6 h) was around 600 °C, although the amorphous phase persists over a range of higher temperatures and the disorder still increases. With a rising calcination temperature, it was found that the solubility of Si and Al is decreasing. However, around 1000 °C, the recrystallization of Al phases enhances the Si solubility again. Yet, for longer dissolution times (1-to-7 d), the dissolved amount of Al and Si is quite similar for calcination temperatures between 600 and 1000 °C. These observations are in accordance with He et al. [46], who found no significant change in mortar compressive strength for kaolinite calcined between 650–950 °C. In contrast, Trümer [21], de Gutierrez et al. [92], and Chakchouk et al. [84] found a slightly rising trend of relative compressive strength and compressive strength, respectively, with calcination temperatures from 600 to 800 °C for kaolinitic clays and processed kaolin, respectively. Rashad [8] summarized from extensive literature research that the optimum calcination temperature differs in the range of 600 to 850 °C, with optimum calcination times between 1–12 h.

It can be summarized that after complete dehydroxylation at around 600 °C, a high reactivity can be achieved and kaolinite is relatively insensitive to higher temperature treatments up to >900 °C. However, regarding slight effects on reactivity with rising temperature, there is no consensus in the literature.

##### Montmorillonite

According to the study by Trümer [21], the dehydroxylation of montmorillonite occurs around 650 °C in the case of the Ca- and around 700 °C in the case of Na–montmorillonite. Other authors found that it occurred at 600–800 °C [20,61,87]. According to Qin et al. [93], the dehydroxylation temperatures decrease with an increasing layer charge of the montmorillonite. Trümer [21] found that dehydroxylation does not lead to any change in the crystal structure as it was observed with kaolinite as XRD reflexes are still persistent after a calcination at 700 °C for Ca- and 800 °C for Na–montmorillonite. After calcination at 800 °C, the Ca–montmorillonite was totally amorphous, and recrystallizations have been observed around 900 and 1000 °C in the form of high-temperature β-quartz, followed by cristobalite, spinell, and anorthite [21]. For Na–montmorillonite, an initial amorphization was observed at a calcination temperature around 800 °C. At around 900 °C, the Na–montmorillonite is totally X-ray amorphous, however, first recrystallizations of spinel and enstatite appear and at around 1000 °C, cristobalite is observed [21,87]. Amorphization and recrystallization occur together. Garg and Skibsted [18] found with NMR studies that amorphization of their investigated montmorillonites already began at around 600 °C, and recrystallization of anorthite, diopside, wollastonite, and hematite was found from 900 °C on. This underlines that the optimum calcination temperature is very sensitive to the composition of montmorillonitic clays.

With solubility tests, Trümer [21] found, for Al solubility of montmorillonite, a maximum temperature of 800 °C for all investigated dissolving times in the same order of magnitude. For the Si solubility, the maximum can be found at calcination temperatures of 800 or 900 °C depending on the dissolution time. At higher temperatures than about 900 °C, the dissolution will be hindered by the decreasing specific surface area and recrystallization. At the optimum temperature, a higher Si dissolution than for metakaolinite was observed. However, the Al dissolution was much lower.

In addition, He et al. [87] found the optimum solubility of Si and Al at a calcination temperature of around 830 °C except for the Si solubility of Ca–montmorillonite, which was the highest at around 730 °C. However, the BET surface constantly decreased from about 730 °C [87].

In addition, Hollanders et al. [62] found for both Ca- and Na–montmorillonite, the highest reactivity was at a calcination temperature of around 800 °C.

##### Illite

According to Trümer [21] and Lopez [61], the dehydroxylation of illite occurs mainly between 450 and 700 °C, but no changes in mineralogy are detectable with X-ray diffraction. In addition, He et al. [47] found the dehydroxilation peak at 580 °C without the collapse of the crystal structure. The amorphization begins at around 900 °C [21,82,94]. However, even after calcination at 930 °C for 100 min., 17 w.% from original 83 w.% of illite could still be detected with XRD by He et al. [47]. For another less pure illitic clay, a partial amorphization of illite was already observed at around 800 °C [21]. In contrast to kaolinite and similar to montmorillonite, the amorphization of illite occurs mainly via sintering and glass formation what can be derived from a decreasing BET surface and SEM investigations. Around 1000 °C spinel and hematite can recrystallize [21]. However, recrystallization phases also depend on the impurities in the investigated clay.

The conservation of the crystal structure of illite and montmorillonite probably results from the smaller amount of crystal water in comparison to kaolinite [20].

For illite, the solubility of Al and Si is much less than for kaolinite and montmorillonite. Trümer [21] found with solubility tests that the longer the dissolution time, the more Al and Si dissolved. In addition, the solubility rises until a calcination temperature of around 900 °C. The Al solubility then decreases, whereas the Si solubility still rises for longer dissolution times (1-to-7 d). With a dissolution time of 6 h, the maximum Si dissolution is also around a calcination temperature of 900 °C. For a calcination at around 1000 °C, the dissolution is slower because of the lower specific surface area caused by sintering. The BET surface area is initially relatively constant (until around 800 °C) and drops from around 20 m^2^/g to <1 m^2^/g already at 900 °C [21].

It can be summarized that the availability of Al and Si in the first hours of the dissolution experiments strongly depends on the specific surface area of the sample.

The temperature-dependent processes during calcination for the different clay minerals are summarized in a simplified form in Table 2. However, the optimum temperature for each clay phase is strongly dependent on its composition.

A good compromise for mixed clays can be expected in the range of 850-to-900 °C.

#### 4.2.3. Cooling

In the literature, only a slight increase in reactivity was found for air-quenched kaolinitic and smectitic clays regarding a calcination temperature range of 500-to-1000 °C when comparing it to the same material slowly cooled down in the furnace [22]. Also, for water-quenched calcined clay (illite/kaolinite) from a calcination temperature of 800 °C, there was only a slight increase in reactivity in comparison to slower cooling in air [95].

It can be assumed that the effect of the cooling rate would be more pronounced at higher proportions of melted phases due to the fixation of the metastable glass state and the inhibition of recrystallization with quenching.

### 4.3. Characteristics of the Calcined Material

#### 4.3.1. Physical Properties

The specific surface area significantly influences the reactivity [86,96]. As with every SCM, the particle morphology and grain-size distribution determine their effectiveness as filler and seed substrates for reaction products [40]. During calcination, the BET surface decreases, and the particle size increases significantly for montmorillonite from about 700 °C and for illite from about 700-to-800 °C, whereas they are quite constant for kaolinite [21,61]. However, this parameter can be adjusted via grinding after calcination.

#### 4.3.2. Amorphous Phase

As the pozzolanic reactivity of calcined clays is defined here by the availability of Si and Al in a cementitious environment, the binding conditions of these elements in the solid phase are decisive. Here, the X-ray amorphous proportion is a first indicator for the reactivity potential [40] since an amorphous phase is a meta-stable solid state in comparison to crystalline Al/Si phases. However, the amorphous content of calcined-mixed clays is not directly linked to the reactivity, as it is known for other SCMs with less variable compositions like blast furnace slag [97]. The chemical composition and atomic structure of the amorphous phase are decisive [96,97]. Based on the discussion in the previous sections, it is evident that the calcination of different clay phases leads to amorphous phases with different reactivity.

As pointed out in Section 4.2.2, metakaolinite is not a real glass, and the high reactivity of calcined kaolinite is based on the exposure of the octahedral layer and lattice stresses induced by the formation of 5-fold coordinated Al after dehydroxilation. In contrast to that, the amorphization of illite and smectite occurs through melting or sintering. Consequently, the insights for glass phases might be transferred to the metaphases of these minerals. Referring to the network hypothesis based on Zachariasen [98] and Warren [99], for (calcium) aluminosilicate glasses, the degree of polymerization and network connectivity significantly influences the reactivity [100,101,102]. The inclusion of most alkali metals and alkaline earth metals decreases the degree of internal order. They act as network modifiers and charge-balancing ions in the network. Si^4+^ is the only exclusive network-forming component in these glasses, whereas Fe^3+^ and Al^3+^ act as network formers (when the charge is balanced) or as network modifiers. In addition, Mg^2+^ can fulfill both the function of network formers such as Al^3+^ cations, and the function of modifiers such as Ca^2+^ cations [103]. The effect of the different cations is reflected in the lower reactivity of Si-rich fly ash compared to Ca-rich fly ash, and the blast furnace slag glasses as the content of network modifiers increases from the first to the latter. Schöler et al. [102] and Kucharczyk [104] found higher quantities of CaO; they also found that Al_2_O_3_ increased the reactivity of the glasses. Durdziński et al. showed that the release of Si from model glasses in an alkali solution increases from silicate glass over aluminum–silicate and calcium–silicate glass to calcium–aluminum glass [105]. However, when comparing the general chemical composition of illites and smectites, based on the chemical composition, the high difference in reactivity cannot be explained. The lower reactivity of illite seems to be related to its higher temperature stability.

Garg and Skibsted found with NMR investigations that during the amorphization of the layer structure of the clays, a number of different SiO_4_ environments occur, and with rising calcination temperature, the reactivity of the clay is lowered again due to the condensation of the SiO_4_ tetrahedrons to an inert 3D-network structure (Q^4^ tetrahedra) [18,19]. When recrystallizations occur, the reactivity is lowered again as these Al/Si-containing phases are considered to be inert or lower reactive in the cement system (see Section 4.2.2). However, as the researchers usually search for the optimum calcination temperature for reactivity, no solid data were found on the behavior of “overburned” clays in a cementitious environment.

## 5. Quantitative Data Compilation and Evaluation

### 5.1. Approach

The reactivity-determining factors of calcined clays were identified in Section 4. In this chapter, a data compilation was performed in order to derive general rules for the potential of the pozzolanic reactivity of clays. It was intended to correlate clay characterization data with reactivity data.

The evaluation regarding the use in alternative binder systems, such as geopolymers, is not considered here. Moreover, the investigations of artificial calcined-clay limestone mixes are not considered here as these mixes can only be compared among each other and are not comparable to the clays alone. Nevertheless, more than 200 scientific publications were found that assess the reactivity of mostly low-grade clays for use as SCM in the cement or concrete industry.

As the reactivity is determined by the composition and treatment of the material, relevant papers should ideally contain a quantitative chemical and mineralogical characterization of the raw and calcined material. In addition, the calcination parameters include furnace type, grain size, calcination temperature, and retention time. These factors are important to understand the change in phase composition and the effect on reactivity. To allow for the comparison of the results, the same reactivity tests and knowledge about the specific surface area and grain size distribution of the calcined clay prior to the experimental program is important. Since publications with all of these characterization data can hardly be found, in a first step, only publications that contain at least a complete quantitative phase analysis of the raw or calcined material were considered. Fifty-five publications with a quantitative phase analysis (of the raw or calcined clay) were identified.

In the literature, the provided information of clay characteristics, as well as the treatment and methods to evaluate the reactivity, strongly differs between the publications. However, as the relative compressive strength is the most frequently used method and other tests are designed to correlate/predict the strength development, publications evaluating the clay using relative compressive strength are considered here. In addition, the few publications using the R^3^ test were considered, as well as some own investigations using the R^3^ test on calcined clays as this is the most promising test for future application. The data from these publications are summarized in the electronic File S1.

When performing relative compressive strength tests with mortars composed of the same raw materials and with the same composition, there is already known to be a discernible standard deviation of the results. However, difficulties in the comparability of the data increase due to the use of different raw materials. The comparability further decreases when the basic composition of the mortars also varies between the different studies. Besides mortars according to EN 196, mortars according to ASTM C311 [106] or their own formulations were also applied in literature studies. In addition, the cement substitution levels used vary from 5 to 40 wt.% between the studies. Furthermore, for mortars using the same basic recipe, the specimen geometry can vary (prisms, cubes, and cylinders). Since there is such a huge variation of boundary conditions in the literature, for the data analysis in Section 5.2, the data were further thinned out to reach more comparability. However, a compromise was aimed to retain multiple datasets for statistical evaluation. Therefore, only compressive strength datasets were evaluated with cement substitution levels of 20–30 wt.% using CEM I with w/b ratios of 0.5. For samples that were calcined at different temperatures, only the temperature with the best relative compressive strength results was considered.

The approach and the potential errors are discussed in more detail in the electronic File S2.

### 5.2. Evaluation

**Figure 4:**
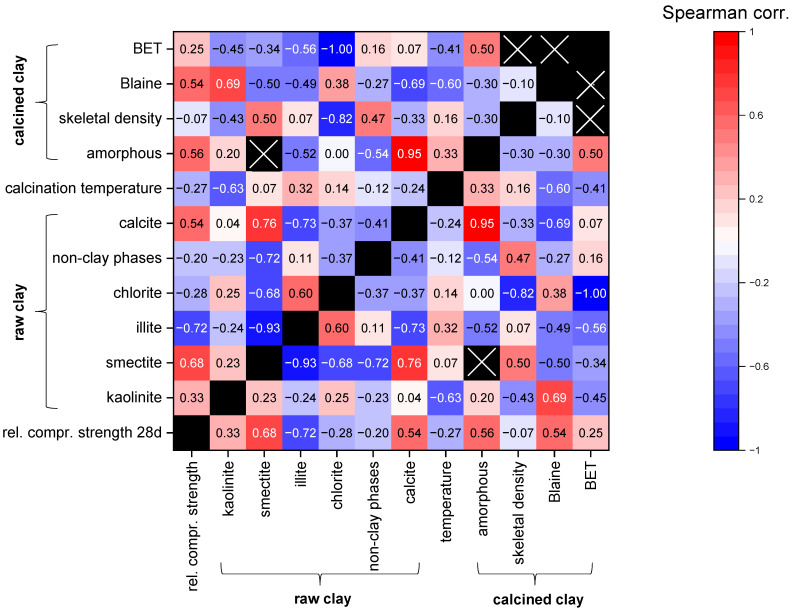
Heat map of Spearman correlations for different parameters possibly influencing reactivity (relative compressive strength data for cement substitution levels of 20–40 wt.% are considered together; numbers in the fields: Spearman correlation coefficients; black fields: n/a; black fields with cross: two or less data points).

Using the defined criteria in Section 5.1, n = 66 datasets of clays are available. Since all datasets do not show normal distributions, the Spearman correlation coefficients for different parameters possibly influencing the reactivity were calculated and are illustrated with a heat-map diagram in Figure 4. The focus of this statistical analysis is intended to be on the influence of the parameters on the relative compressive strength, but the correlation coefficients of the other combinations are also presented for the overview. However, the data should be interpreted carefully, as for many parameters, not a lot of data are available due to the differences in composition and characterization (see Table 3). In addition, the combinations of some parameters were not intended to show any correlations with each other such as the different mineral phase contents. For example, calcite positively correlates with smectite, which is obviously a random trend since few data originate from smectitic marl.

For a uniform rating of the correlation strength, the wording corresponding to Table 4 was used.

Figure 4 shows that the content of the most reactive phases in the raw clay, kaolinite and smectite, display a weak and strong positive correlation, respectively, with the increasing relative compressive strength. Although calcined illite is more reactive than inert components, its increasing content is correlated to decreasing relative compressive strength (rs = −0.72) as the remaining proportion, which may contain components with higher reactivity (kaolinite and smectite), is decreasing. A similar trend can be expected for non-clay phases. However, there is only a weak trend (rs = −0.20). In addition, for the four-layer clay-mineral chlorite in the raw clay, no positive effect on the relative compressive strength can be observed (rs = −0.28). A possible effect might be superimposed by the other clay phases since the chlorite content only reached up to 22 wt.% in this study, and only few data are available. Moreover, it is not clear whether chlorite is properly amorphized, since studies quantifying both raw clay and calcined clay phase composition are rare. The amorphous content of the calcined clay shows moderate correlation with relative compressive strength (rs = 0.56). The physical parameters also only have a very small database. However, for the Blaine-specific surface area of the calcined clays, a moderate positive correlation to relative compressive strength can be observed (rs = 0.54). When looking on other intercorrelations, higher calcination temperatures are associated with the illite content of the raw material (rs = −0.32), and lower calcination temperatures are associated with the kaolinite content of the raw material (rs = 0.63), which is in accordance with the findings of the qualitative literature evaluation. The results are from studies in which the optimum calcination temperature was searched for and from studies in which the calcination temperature was selected based on experience. Higher calcination temperatures are moderately correlated to lower Blaine-specific surface areas, which might illustrate the sintering/melting effects (rs = −0.60). The calcite content of the raw clay shows a very strong correlation with the amorphous content of the calcined clay (rs = 0.95) and a strong negative correlation with the Blaine-specific surface area (rs = −0.69), which might indicate the lowering of the melting temperature as discussed in Section 4.1.1. A more profound statistical data analysis like multiple regression analysis is not possible since the database is too small for the number of parameters, and not every dataset contains values for each parameter.

**Figure 5:**
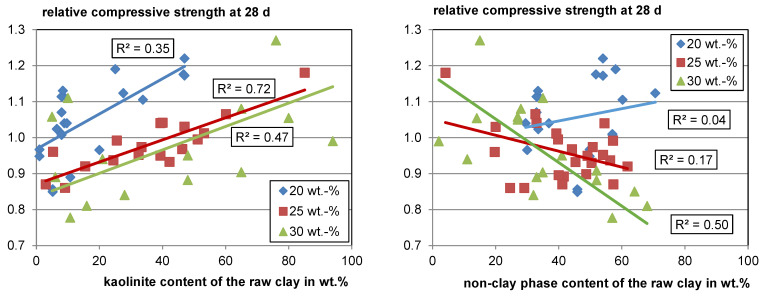
Relative compressive strength as a function of the raw clay-phase contents.

Figure 5 (left) shows the overall trend of rising strength with rising kaolinite content in the raw clay for each substitution level. While the different boundary conditions discussed in Section 5.1 certainly result in significant scattering of the data, it seems evident that the strength contribution is not solely a function of the kaolinite content in the raw clay, since the relative compressive strength using calcined clay varies significantly at comparable kaolinite contents in the raw clay. Bratoev et al. [66] found with the Chapelle test that the montmorillonite content in the raw clay also significantly impacts the reactivity with a Ca(OH)_2_ consumption of about 50 wt.% less than kaolinite.

When looking at the relative compressive strength as a function of the non-clay phases in the raw clay (see Figure 5, right), it becomes visible that for a low substitution level, the content has a low impact on the reactivity, whereas with a rising substitution, a clear trend of decreasing relative compressive strength with increasing content of non-clay phases can be seen. This trend can be explained by the decreasing amount of potentially reactive phases with an increasing amount of non-clay phases. Furthermore, it becomes visible that most of the calcined clays perform well and would meet, for example, the minimum relative compressive strength of 0.75 defined for fly ashes with a cement substitution level of 25 wt.% according to EN 450, even with only about 30 wt.% of total clay phase content in the raw material.

**Figure 6:**
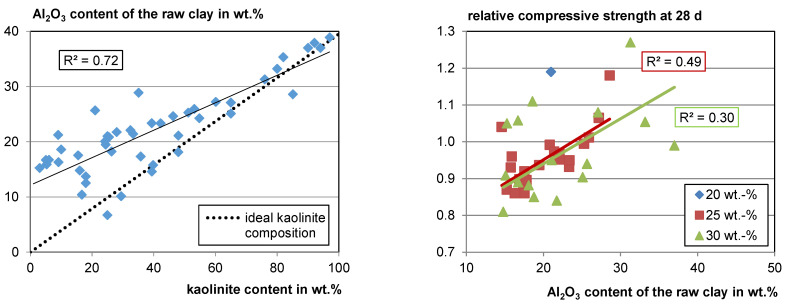
Al_2_O_3_ content versus kaolinite content (**left**) and relative compressive strength versus Al_2_O_3_ content (**right**).

Since Si and Al occur in all of the main clay-mineral phases and in the most frequent non-clay phases such as quarz (Si), feldspars (Al, Si), and mica (Al, Si) it does not seem possible to extract information about the reactivity of mixed clays just from the chemical composition (see Section 4.1.2). However, when looking at the gathered data, there is a strong correlation between Al_2_O_3_ and the kaolinite content in the raw clay (see Figure 6, left). When the kaolinite content is low, the deviation from the ideal kaolinite composition is high as the probability for other Al-bearing phases is high. However, at kaolinite contents higher than about 60 wt.%, the Al_2_O_3_ content of the sample fits the Al_2_O_3_ content of an ideal kaolinite quite well since kaolinite has a comparatively high Al content. This means, in turn, if the Al_2_O_3_ content in clay is higher than about 30 wt.%, a high kaolinite content is probable, as well as a high reactivity potential. Of course, this does not apply if, for example, aluminum ores are included. Since there is a correlation of the Al_2_O_3_ content to the kaolinite content in the raw clay, a slight correlation of the Al_2_O_3_ content with relative compressive strength also becomes visible (see Figure 6, right).

In some cases, the Al_2_O_3_ content is lower than the value calculated for an ideal kaolinite with this content in the raw clay. In these cases, an amorphous phase may exist in the raw clay for which the authors may not have searched with XRD. Apart from low crystalline clay phases, e.g., X-ray amorphous opal, as well as Fe and Al hydroxides, can also occur in the raw clay. In this case, the total kaolinite content would be lower than measured. No clear correlation of other Al-bearing phases with Al_2_O_3_ was observed. However, there are less data available.

**Figure 7:**
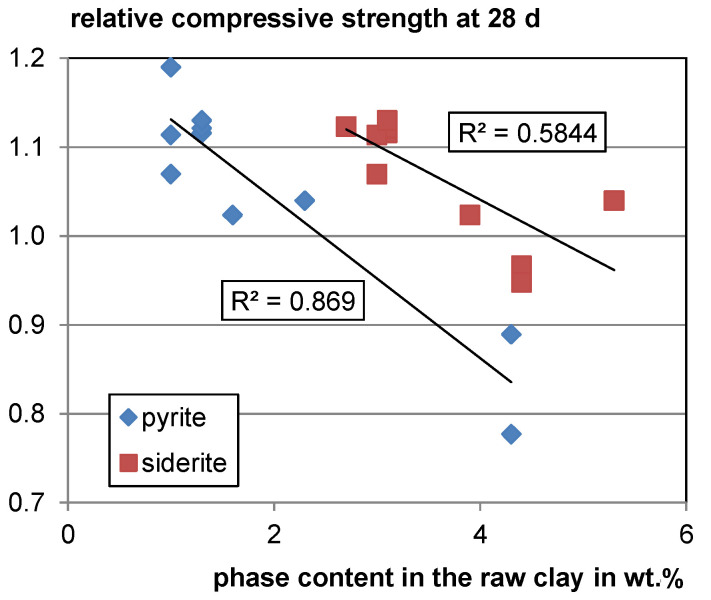
Relative compressive strength as a function of the pyrite and siderite content in the raw clay.

For the decomposing Fe-bearing phases, siderite and pyrite, a moderate and strong negative trend of relative compressive strength with increasing phase contents in the raw clay can be observed (see Figure 7). Both pyrite and siderite decompose at temperatures around 500 °C under atmospheric conditions [107,108] which is lower than the calcination temperature of clays. However, it is not clear whether this is a random trend as there are only few data available, and both phases are assumed to form inert hematite during calcination. For verification, further studies are needed.

**Figure 8:**
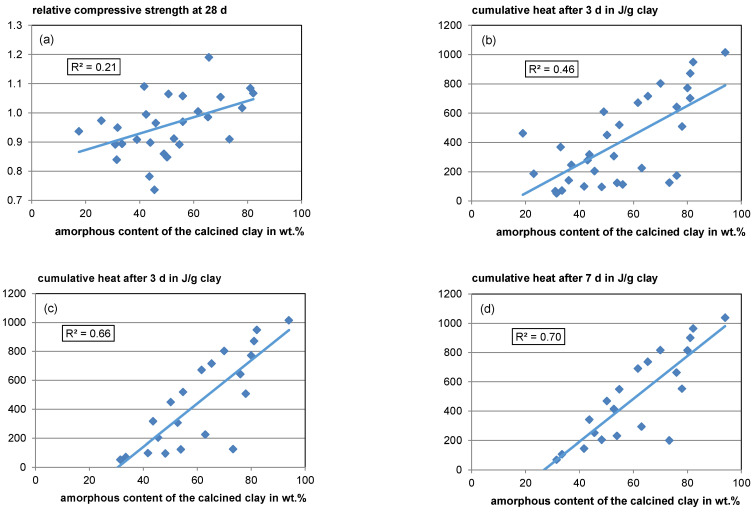
Relative compressive strength (**a**) and cumulative heat release according to ASTM C1897–20 [51] versus amorphous content (**b**): Heat after 3 days for all available data; (**c**): Heat after 3 days for data only available also for 7 days; (**d**): Heat after 7 days).

Apart from inaccuracies resulting from the different boundary conditions of the datasets, regarding the amorphous content, different types of amorphous phases with different reactivities resulting from the corresponding raw clay phases make it difficult to correlate amorphous content with reactivity (see Section 4.1.1 and Section 4.3.2). However, a weak overall trend of rising relative compressive strength with rising amorphous content can be found with a coefficient of determination (R^2^) of 0.21 (see Figure 8, top left).

For the cumulative heat release after 3 days in the R^3^ test (ASTM C1897-20 [51]), a moderate overall trend with rising amorphous content is visible (see Figure 8, top right). However, the scatter is also high with an R^2^ of 0.46. After 7 days, the correlation seems to be better with an R^2^ value of 0.7 (see Figure 8, bottom left). However, since in some literature studies the measurement only takes 3 days, fewer data are available for the 7-day heat. When analyzing just the same data available for the 7-day heat regarding its 3-day heat, the R^2^ is quite similar (R^2^ = 0.66), indicating that a 3-day measurement is sufficient (see Figure 8, bottom left) since calcined clays react comparatively fast. Unfortunately, for most of the R^3^ test data, no phase composition of the raw material is available allowing no profound interpretation concerning the effects of raw clay composition.

## 6. Conclusions and Outlook

The literature research confirms that most of the “low-grade” clays are suitable for use as SCM. However, their potential applications as SCM depend on their actual performances, which can differ significantly between the different clays.

The following main parameters were identified to have impact on the reactivity potential:

- Phase composition of the raw material: The kaolinite content is the most important factor for the reactivity potential of the clays. However, montmorillonite is also known to have a significant reactivity potential. Illite has the lowest reactivity potential of the main clay phases. Calcite is known to react with alumina from the clays, forming carbo–aluminate hydrates that contribute to strength development. When calcining calcite and dolomite together with clay minerals, the reaction products can lower the melting point, build glassy phases and lower the recrystallization temperature. Other phases show minor effects on the reactivity.
- Calcination parameters: The furnace type due to different dispersion, the grain-size distribution prior to calcination due to different effectiveness of calcination progress depending on the surface-to-volume ratio, the calcination temperature due to the influence on the structural changes of the clay minerals, and the retention time are decisive for reactivity development. However, for the different types of clay-mineral phases, the parameters have different optima. In addition, for the same clay-mineral phase, the optima depend on their actual composition and crystallinity.
- Composition of the calcined material: The particle-size distribution/specific surface area prior to application are important as the surface area determines the reaction kinetics. The phase composition of the raw material and the calcination parameters determine the phase composition of the calcined material. Here, the reactivity of the amorphous phase is strongly dependent on the phase composition of the raw material.

When compiling data from the literature with the aim to derive significant correlations between the sample characteristics and reactivity, it was found that the characterization of the materials is mostly insufficient, and the assessment methods for reactivity strongly differ between publications. Therefore, the different data can hardly be compared with each other. However, it has been shown that despite inaccuracies due to different boundary conditions, the overall trends found with qualitative literature analysis could be confirmed, indicating that with more comparable data, good prediction models seem possible. With this publication, a roadmap is provided, showing which properties are relevant and required not only to make a good assessment of the available clay, but also to provide comparable results to enable linking to other studies in order to advance the research on this subject.

## Figures and Tables

**Table 1:** Compilation of test methods to assess the reactivity of SCMs.

Measured Parameter	Test Reference	Applied e.g., in	Comment
Portlandite consumption	Chapelle test [32]	[33]	No correlation to the 28 days relative compressive strength and a bad reproducibility
	Modified Chapelle test (NF P18-513 [34])		Good correlation to the 28 days relative compressive strength for pozzolanic SCMs and moderate reproducibility
	The Frattini test (EN 196-5 [35])		Good correlation to the 28 days relative compressive strength for pozzolanic SCMs and bad reproducibility caused by the use of different cements
	The Indian standard lime reactivity test (IS 1727 [36])		Moderate correlation to the 28 days relative compressive strength and good correlation for 90 days
	Diverse other variations	[37,38,39,40]	-
Ion solubility (Si)	Reactive silica test (EN 196-2 [41] and EN 197-1 [42])	[33,43]	Bad correlation to relative strength
	Method of Surana and Josh [44]	[43,44,45,46,47]	Good correlation of the dissolved Si with mortar compressive strength
Ion solubility (Si and Al)	Method of Buchwald et al. [48]	[21,48,49]	-
Ion solubility	Diverse other variations	[22,50]	-
Reaction heat/bound water	R^3^ test (ASTM C1897-20) [51]	[33,52]	Good correlation to the 28 days relative compressive strength and moderate reproducibility
Reaction heat	Diverse variations	[53,54,55,56,57]	-
Electric conductivity	Diverse variations	[58,59]	-
Relative compressive strength	Diverse variations with cement paste, mortar, and concrete	Numerous studies	Often used as benchmark for reactivity; results strongly depending on the recipe

**Table 2:** Processes during calcination summarized from literature.

Process	Kaolinite	Montmorillonite	Illite
	Temperature in °C		
Dehydroxylation	~450–700	~600–800	~450–700
Amorphization		~800–900	~900
Beginning sintering/melting	-	~900	
Beginning recrystallization	~925		~1000

**Table 3:** Number of parameters (n) available for statistical analysis (see Figure 4).

Parameter		n
Relative mortar compressive strength at 28 d		66
Content in the raw clay	Kaolinite	55
	Smectite *	24
	Illite	55
	Chlorite	16
	Non-clay phases	66
	Calcite	24
Applied/optimum calcination temperature		64
Calcined clay	Amorphous content	14
	Skeletal density	13
	Blaine	20
	BET	11

* In most cases montmorillonite but not specified in every publication.

**Table 4:** Language use for rating the correlation regarding Spearmann and determination coefficients.

Coefficient Range	Rating
0.2–0.4	Weak
0.4–0.6	Moderate
0.6–0.8	Strong
0.8–1.0	Very strong

## Data Availability

Data are contained within the article and supplementary materials.

## Supplementary Materials

The following supporting information can be downloaded at: https://www.mdpi.com/article/10.3390/ma17020312/s1, File S1: Database; File S2: Detailed discussion of the evaluation approach. References [109,110,111,112,113,114,115,116,117,118,119,120,121,122,123,124,125,126,127] are cited in the supplementary materials.
