# Supplementary material for: Reactivity of Calcined Clays as SCM—A Review
Source: Materials (Basel). 2024 Jan 8;17(2):312. doi: 10.3390/ma17020312 (PMC10820630; doi:10.3390/ma17020312)
Supplement: Supplementary file 1 [file materials-17-00312-s001.zip › File S2.pdf]

1 Approach of the quantitative data analysis

The reactivity-determining factors of calcined clays were identified in chapter 3. In this chapter, a data compilation was done in order to derive general rules for the potential of the pozzolanic reactivity of clays. It was intended to correlate clay characterization data with reactivity data. Due to the high variation in the composition of clays and the not precisely understood interdependencies of the reactivity-determining factors there are many studies that assess the potential of the clays in each individual case. So, the data was taken from publications that assess the reactivity of mostly low-grade clays for the use as SCM in the cement or concrete industry with methods described in chapter 2.

The evaluation regarding the use in alternative binder systems, like geopolymers, is not considered here. Moreover, the investigations of artificial calcined clay lime stone mixes are not considered here as these mixes can only be compared among each other and are not comparable to the clays alone. Concerning the resulting synergies we refer the reader to the most recent reviews on this subject, e.g. [70] . Nevertheless, more than 200 scientific publications were found dealing with the specified topic.

As the reactivity is determined by the composition and treatment of the material, relevant papers should ideally contain a quantitative chemical and mineralogical characterization of the raw and calcined material and, in addition, the calcination parameters like furnace type, grain size, calcination temperature and retention time. These factors are important to understand the change in phase composition and the effect on reactivity. To allow the comparison of results, the same reactivity tests and knowledge about the specific surface area and grain size distribution of the calcined clay prior to the experimental program is important.

Since publications with all of these characterization data can hardly be found, in a first step, only publications that contain at least a complete quantitative phase analysis of the raw or calcined material were considered. In a few publications, the content of kaolinite or other clay minerals was quantified using TGA; these data were also not considered, as no quantification of the other components is given. More detailed information on the ordering conditions or the

composition of the clay minerals by means of IR, Raman or NMR spectroscopy, if available, was also not considered; instead, the clay minerals were divided into simple groups (kaolinite, illite, smectite, chlorite) in order to generate a comparable database. 55 publications with a quantitative phase analysis (of the raw or calcined clay) were identified.

In Table S1, the important parameters and characteristics that determine reactivity and the main reactivity evaluation methods are listed with the number of publications that provide the corresponding data. It becomes clear that the provided information of clay characteristics and treatment is strongly differing between the publications. Even in the same category the data are not necessarily comparable due to different specifications or experimental set ups. Above all, the methods to evaluate the reactivity are strongly differing between the publications. However, as the relative compressive strength is the most frequently used method and other tests are designed to correlate / predict the strength development, publications evaluating the clay using relative compressive strength are considered here. Also, the few publications using the R^3 test were considered as well as some own investigations using the R^3 test on calcined clays as this is the most promising test for future application. The data from these publications are summarized in the electronic appendix.

Table S1. Important characterization parameters and number of publications containing these data using 55 publications with quantitative phase analysis.

characterization			number of publications
quantitative phase analysis of the raw material			52
quantitative phase analysis of the calcined material			7
calcination parameters	calciner type		37
	grainsize / specific surface		31
	temperature		52
	duration		43
calcined material before experimental program	particle size distribution		22
	specific surface		32
	quantitative phase analysis		11
performance test	relative compressive strength	paste	11
		mortar	37
		concrete	3
	R^3 heat calorimetry		4
	Frattini		8
	Chapelle test		3
	other versions of portlandite consumption		9
	different versions of ion solubility		14

When performing relative compressive strength tests with mortars made of the same raw materials and with the same composition there is known to be already a discernible standard deviation of the results. However, difficulties in comparability of the data increase by using different raw materials. The comparability further decreases when also the basic composition of the mortars is varying between the different studies. Beside mortars according to EN 196 also mortars according to ASTM C311 or own formulations were applied. Also, the cement substitution levels used vary from 5 to 40 wt.-% between the studies. Furthermore, for mortars using the same basic recipe, the specimen geometry can vary (prisms, cubes, cylinders). Data from mortars with insufficiently specified compositions are not considered in the electronic appendix as well as specially conditioned mortars which allow no comparison. Because there is such a huge variation of boundary conditions in literature, for the data analysis in Chapter 4.2, the data were further thinned out to reach more comparability; however, a compromise was aimed for to still have multiple datasets for statistical evaluation. Therefore, only compressive strength datasets were evaluated with cement substitution levels of 20-30 wt.-% using CEM I with w/b ratios of 0.5. For samples that were calcined at different temperatures only the temperature with the best relative compressive strength results was considered.

It has to be kept in mind that differences in the specific surface area / grain size distribution further lead to inaccuracies in comparability when trying to identify the strength contribution of different clay phases. BET values are usually much higher than Blaine values for calcined clays due to their porous structure which is not captured with the Blaine method. Furthermore, not for all samples data of the specific surface area and / or grain size distribution is available.

Furthermore, the observational error of the quantitative determination of the phase composition cannot be estimated. In most of the cases, no amorphous content was given for the raw material. However, also raw clay can contain X-ray amorphous phases.
